# Restless legs syndrome and growing pains in childhood: understanding the link

**DOI:** 10.3389/fneur.2025.1603694

**Published:** 2025-08-22

**Authors:** Alexandra E. V. Rosen, Fabiana Ursitti, Nausica Stella, Laura Papetti, Martina Proietti Checchi, Alessandra Voci, Luigi Mazzone, Massimiliano Valeriani, Romina Moavero

**Affiliations:** ^1^Unit of Child Neurology and Psychiatry, Department of Systems Medicine, Tor Vergata University of Rome, Rome, Italy; ^2^Unit of Developmental Neurology, Bambino Gesù Children’s Hospital, IRCCS, Rome, Italy; ^3^Unit of Child Neurology and Psychiatry, Department of Wellbeing of Mental and Neurological, Dental, and Sensory Organ Health, Policlinico Tor Vergata Hospital, Rome, Italy; ^4^Translational Pain Neuroscience and Precision Medicine, CNAP, Department of Health Science and Technology, School of Medicine, Aalborg University, Copenhagen, Denmark

**Keywords:** restless legs syndrome, growing pains, children, Willis-Ekbom disease, migraine equivalents, sleep disorders, migraine, leg pain

## Abstract

**Introduction:**

Restless Legs Syndrome (RLS), known as Willis–Ekbom disease, is a common neurological condition that often goes undiagnosed, especially in children. Characterized by an irresistible urge to move the legs, it is typically more pronounced in the evening and at rest. Growing Pains (GP), common in childhood and associated with migraine, present apparently overlapping symptoms with RLS, making it sometimes difficult to distinguish between the two. Understanding their relationship is important to make correct diagnosis and treatment.

**Methods:**

We performed a literature review on PubMed using combinations of terms such as “Restless Legs Syndrome,” “Growing Pains,” and “children” to explore diagnostic criteria and the relationship between RLS and GP. Studies included those from 2000 to 2024 that involved individuals under 18 with diagnosis of RLS or GP, written in English language.

**Results:**

The 24 studies we included in our analysis showed that RLS and GP share common physiological factors, including serotonin dysfunction, iron deficiency, and low vitamin D levels. Evidence from genetic studies suggests a familial link in the development of both conditions. These findings suggest that GP might be an early form of RLS. Both conditions are linked with headaches, sleep disorders, and neuropsychiatric conditions like ADHD. Treatment for both conditions includes iron supplements, dopamine agonists, and non-medical approaches such as stretching or physical exercise. Our narrative review shows that, though distinct, RLS and GP might share common underlying causes.

**Discussion:**

RLS and GP are common in pediatric populations, but diagnosis can be challenging due to symptom overlap. This review offers an updated and integrative framework for understanding RLS and GP, highlighting the need for more specific, evidence-based diagnostic criteria. Further research is needed in order to clarify their relationship, refine diagnostic criteria, and explore their genetic and neurobiological mechanisms.

## 1 Introduction

Restless legs syndrome (RLS), known as Willis–Ekbom disease, is a common yet underdiagnosed neurologic condition affecting also school-aged children and adolescents (1). The first formal diagnostic criteria for RLS were defined in 1995 by the International Restless Legs Syndrome Study Group (IRLSSG), with a revision specific to pediatric cases introduced in 2012 (2). The aforesaid criteria are listed in Table 1.

**Table 1:** Restless legs syndrome diagnostic criteria.

Restless legs syndrome diagnostic criteria
An urge to move the legs usually but not always accompanied by or felt to be caused by uncomfortable and unpleasant sensations in the legs
The urge to move the legs and any accompanying unpleasant sensations begin or worsen during periods of rest or inactivity such as lying down or sitting
The urge to move the legs and any accompanying unpleasant sensations are partially or totally relieved by movement, such as walking or stretching, at least as long as the activity continues
The urge to move the legs and any accompanying unpleasant sensations during rest or inactivity only occur or are worse in the evening or night than during the day
The occurrences of the above features are not solely accounted for as symptoms primary to another medical or behavioral condition (e.g., myalgia, venous stasis, leg edema, arthritis, leg cramps, positional discomfort, habitual foot tapping)
Specifiers
Specifiers for clinical course Chronic-persistent RLS/WED: symptoms when not treated would occur on average at least twice weekly for the past year. Intermittent RLS/WED: symptoms when not treated would occur on average Specifier for clinical significance The symptoms of RLS/WED cause significant distress or impairment in social, occupational, educational or other important areas of functioning by their impact on sleep, energy/vitality, daily activities, behavior, cognition or mood.
Features supporting the diagnosis of RLS/WED Periodic leg movements Dopaminergic treatment response Family history of RLS/WED among first-degree relatives Lack of expected daytime sleepiness
Features to be considered for a comprehensive diagnostic assessment of RLS/WED Gender Age of onset History of the course of the disease Sleep disturbance Degree of pain versus discomfort Parts of the body involved Daily pattern of symptoms and activity levels History of pregnancy History of iron deficiency

RLS is characterized by an irresistible urge to move the legs, usually but not always accompanied by pain and/or unpleasant sensations. Symptoms typically occur at rest, are relieved by movement, and predominantly manifest in the evening or at night. Although the legs are most commonly affected, some patients may also experience symptoms in other body regions, including the arms, face, abdomen, or even the genital area (3). The severity of RLS varies widely, with most patients experiencing mild to moderate symptoms, while 1–3% report severe and frequent episodes.

Despite the availability of standardized diagnostic criteria, RLS is frequently misdiagnosed in childhood, and one of the most common differential diagnoses is growing pains (GPs). GPs are considered as part of childhood migraine syndrome, alongside conditions such as cyclic vomiting syndrome, abdominal migraine, benign paroxysmal vertigo, and benign paroxysmal torticollis (4, 5).

The earliest reference to growing pains (GPs) in the medical literature dates back to 1823, when Duchamp described muscular aches and pains occurring in children around puberty, attributing them to the process of growth (6). However, his observations lacked precise diagnostic criteria, leaving uncertainties regarding the exact nature of these pains, their anatomical distribution, and their underlying pathophysiology.

A more detailed consideration of the relationship between GPs and other conditions emerged in 1975, when Ekbom (7) described a case of a patient whose childhood GPs persisted into adulthood and later evolved into RLS. He noted that the discomfort experienced during childhood differed from the symptoms of RLS in adulthood, and that in other family members, GPs resolved spontaneously without progressing to RLS. Based on these observations, Ekbom concluded that GPs and RLS were distinct clinical entities. However, he also acknowledged that some patients with childhood-onset RLS had been misdiagnosed as having GPs, suggesting a potential overlap in their early manifestations. GPs are considered migraine equivalents due to their shared characteristics with migraine, especially in children, with some patients experiencing migraine associated symptoms (nausea, anorexia and abdominal pain) and also having a family history of migraine. Furthermore, migraine preventive treatments are effective for GPs, highlighting that both conditions share similar pathophysiological mechanisms (8).

**Figure 1:**
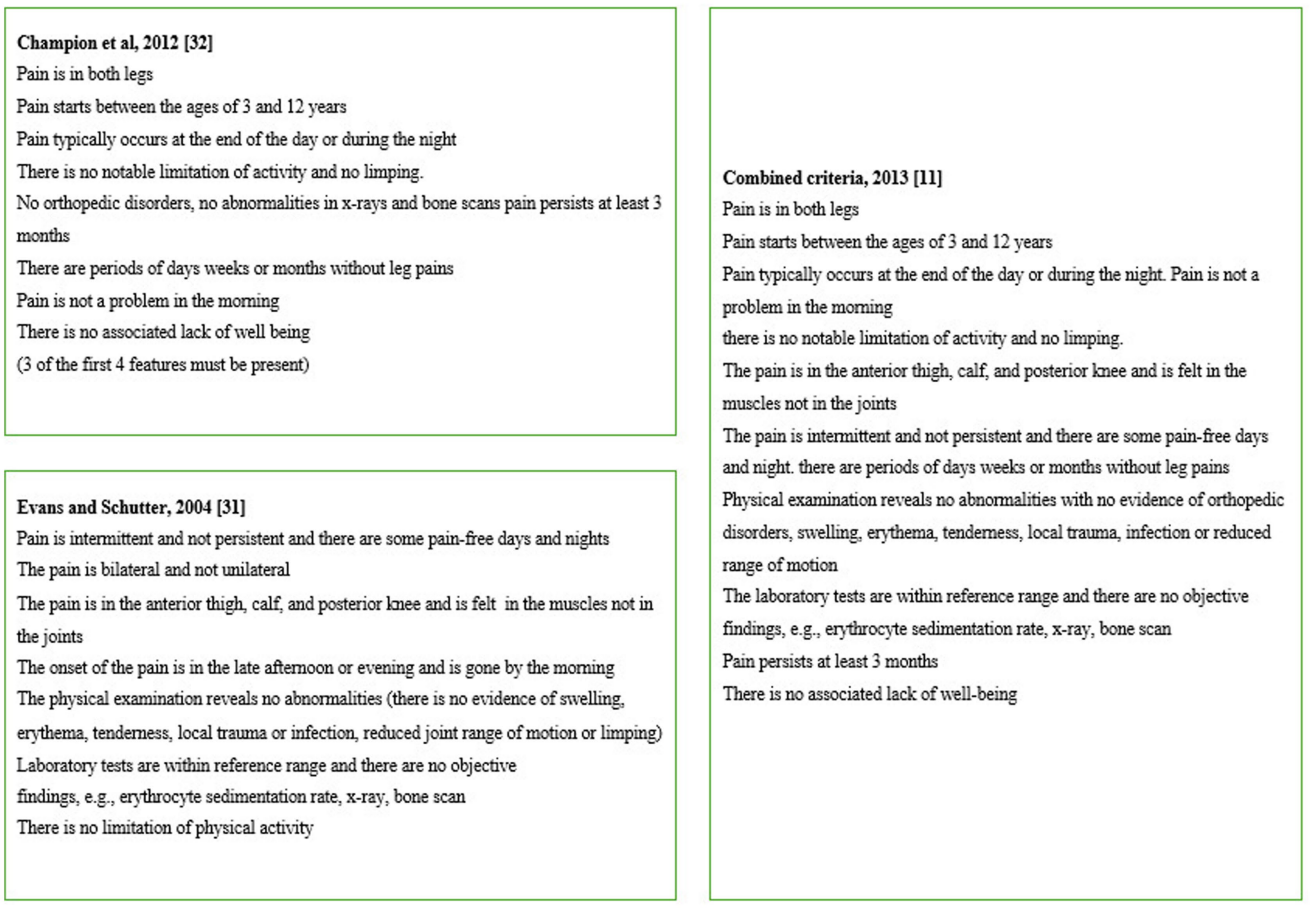
Growing pains features.

Unlike RLS, which benefits from well-defined diagnostic criteria and a clear clinical profile, GP remains a largely descriptive entity, lacking universally accepted diagnosis standards. Attempts to formalize a diagnostic framework have been made by different authors, including Evans and Scutter (9) and Champion et al. (10), whose criteria were later integrated into a single system in 2013 (11) (Figure 1). Notably, many of the diagnostic features of GPs overlap with those of RLS, with two key exceptions: GPs are classically bilateral, whereas RLS can be unilateral or bilateral, and GPs are exclusively painful, while RLS is associated with a broader spectrum of sensory discomfort, including tingling, restlessness, and an urge to move (2).

Both RLS and GPs are common in pediatric populations, with definite RLS diagnosed in approximately 1.9% of children aged 8–11 years and 2% of those aged 12–17 years. There is no gender preference till late adolescence, with a female preponderance beyond that (12). The prevalence of GPs varies widely across studies, but conservative estimates suggest a prevalence of around 4.7% (11). Given their frequency and symptomatic overlap, understanding the relationship between these two conditions is of clinical relevance.

The clinical overlap between RLS and GP contributes to the persistent difficulty in establishing a clear diagnostic framework for GP. This raises the question of whether GP represents a distinct condition or part of a broader spectrum of pediatric movement or pain disorders. Clarifying these distinctions is essential to improve diagnostic accuracy and guide appropriate management strategies.

This narrative review aims to critically analyze the existing literature on restless legs syndrome and growing pains to determine whether they represent distinct disorders or different manifestations of a shared pathophysiological process at different life stages. By examining clinical features, comorbidities, genetic findings, neurophysiological mechanisms, and treatment responses, we seek to identify areas of overlap and divergence, and ultimately assess whether current evidence supports a definitive differentiation or highlights the need for further investigation.

## 2 Materials and methods

This narrative review was conducted in accordance with the SANRA (Scale for the Assessment of Narrative Review Articles) guidelines to ensure methodological quality and transparency (13).

A literature search on PubMed was performed.

A variety of terms were arbitrarily selected and employed for the search: [restless legs syndrome AND/OR growing pains] AND [children OR childhood] OR [restless legs syndrome AND/OR growing pains] AND diagnostic criteria. These terms uncovered more articles relevant to achieving the *a priori* goals of determining the present status of diagnostic criteria for growing pains and restless legs syndrome and evaluating the current literature exploring the relationship between growing pains and restless legs syndrome.

In the first stage, the studies were selected according to the following occurrences:

- Involved individuals with RLS and GPs;
- Involved children and adolescents up to 18 years of age;
- Reported the diagnostic evaluation methods for RLS and GPs;
- English language;
- Published from January 2000 to January 2024.

Articles published before January 2000 and not written in English language were not included in the review except when their historical importance was evident.

Furthermore, to ensure comprehensive coverage of the existing literature, the reference lists of all selected articles were manually reviewed to identify any additional relevant studies that may have been missed in the initial search.

## 3 Results

**Figure 2:**
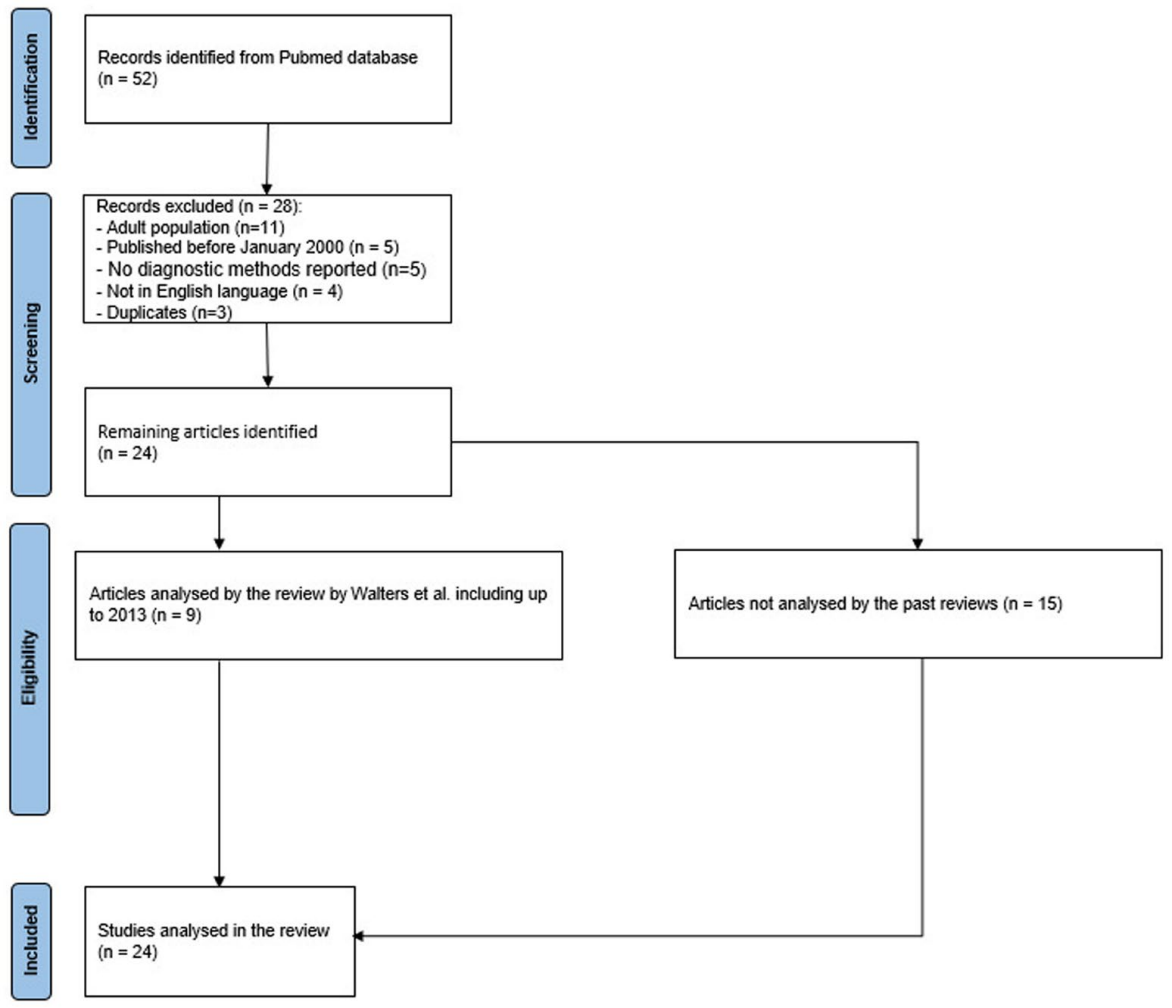
Inclusion process.

A total of 52 articles were found, 5 of which by citation matching. We excluded 28 articles (due to language, publication date, or methodological issues, see Figure 2) and analyzed the remaining 24.

### 3.1 Pathophysiology of RLS and GP

Several studies have explored the pathophysiological mechanisms underlying RLS and GP, with particular attention to the role of serotonin, iron metabolism, and vitamin D.

Serotonin dysfunction has been implicated in both conditions. In 1992, Sandyk (14) first reported that serotonin (5-HT) is a key neurotransmitter involved in multiple central nervous system functions, and its dysregulation is linked to several neurological and neuropsychiatric disorders, including RLS. In the context of RLS, some studies suggest that altered serotonin levels may interfere with dopaminergic pathways, particularly in the basal ganglia, where both systems interact. Increased serotonergic activity has been hypothesized to reduce dopamine availability, potentially worsening RLS symptoms (15). Moreover, selective serotonin reuptake inhibitors (SSRIs), which increase extracellular serotonin, have been associated with the onset or exacerbation of RLS symptoms in some patients (16). Despite these findings, current pharmacological treatments for RLS primarily target the dopaminergic system, as dopamine agonists consistently demonstrate symptomatic relief, likely due to their action on central dopaminergic circuits involved in motor control and sensory modulation (17, 18). A similar serotonergic involvement has been proposed for growing pains, which share pathophysiological features with the migraine syndrome and its equivalents (19). 5-HT also plays a dual role in migraine pathophysiology, influencing both the brainstem and pain transmission networks, and therapies targeting serotonin receptors, like triptans, can modulate this process to relieve migraine pain (20). Some evidence suggests that altered sensory processing and serotonergic imbalance could link growing pains to pediatric forms of the migraine syndrome, supported by the frequent co-occurrence of migraine in affected children or their families (21). This points to a possible role of serotonin in pain syndromes of developmental age. Further research is needed to clarify whether serotonin-targeting therapies could play a complementary or alternative role in treatment.

Iron metabolism also appears to play a crucial role. Sevindik et al. (22) found that serum ferritin levels were significantly lower in migraine patients with RLS compared to those without RLS. Similarly, Kim et al. (23) reported that pediatric RLS patients had significantly lower ferritin levels than adults, despite remaining within the normal range. Champion et al. (24) identified an association between GP and iron deficiency in multivariable analyses, while Donnelly et al. (25) demonstrated a persistent link between iron deficiency, migraine, persistent pain, and RLS (see also section 3.3.2 for further discussion of migraine associations).

Vitamin D deficiency has also been associated with both conditions. Evans et al. (26) found that hypovitaminosis D was present in 87% of patients with either RLS or GP, suggesting a potential shared metabolic component.

Overall, these findings indicate that serotonin, iron, and vitamin D deficiencies may contribute to the pathogenesis of both RLS and GP, supporting the hypothesis that these conditions may share common underlying mechanisms.

### 3.2 Genetic findings

While no genome-wide association studies (GWAS) have comprehensively examined the genetic basis of RLS and GP, some studies provide insight into their heritability.

Türkdoğan et al. (27) reported that a family history of RLS was present in 14.5% of affected children, whereas GP showed a stronger familial association, with 44.2% of children having a positive family history. This indicates that genetic predisposition may be more pronounced in GP than in pediatric RLS. Champion et al. (28) and Donnelly et al. (25) suggested that RLS with painful symptoms may have a genetic component and is associated with both migraine and non-migraine headaches, as well as recurrent abdominal pain (see section 3.3.2). This points to the possible involvement of genes related to pain modulation and sensory processing in the pathophysiology of RLS.

- Twin studies further support a genetic influence on GP. Champion et al. (24) analyzed twin families and found that GP and a specific GP-related phenotype were not associated with gender. Moreover, monozygotic twin pairs exhibited greater similarity for GP and GP-specific traits compared to dizygotic twin pairs, suggesting a heritability component. Interestingly, the GP-specific phenotype was associated exclusively with maternal painful RLS.

Although specific genetic loci have not been identified for GP, RLS has been associated in adult populations with variants in genes such as *MEIS1*, *BTBD9*, *MAP2K5/SKOR1*, and *PTPRD*, many of which are involved in neural development, iron metabolism, and dopaminergic signaling (29, 30). The absence of such associations in GP suggests that the genetic architecture may differ between the two conditions: while RLS may involve genes linked to neurodevelopment and dopaminergic pathways, GP might involve genes related to pain sensitivity or sleep regulation, although this remains to be confirmed by future studies.

These observations support the hypothesis that, although RLS and GP may share a familial or heritable basis, the underlying genetic mechanisms — or at least the key genes and biological pathways — may not entirely overlap. Further studies, particularly those using molecular genetics or GWAS approaches, are needed to clarify the extent of these differences and to identify specific genetic markers for each condition.

These findings suggest a possible hereditary component in both conditions, though further genetic studies are needed to clarify the underlying mechanisms.

### 3.3 Relationship between restless legs syndrome and growing pains

Most authors agree that RLS and GPs should be considered distinct disorders. For example, Türkdoğan et al. (27) observed that a significant number of patients with RLS and GPs shared clinical features, but only 0.03% of a cohort of 192 children met the diagnostic criteria for both conditions. This minimal overlap indicates that, despite some symptomatic similarities, the coexistence of a combined phenotype is exceptionally uncommon. Therefore, the shared features probably reflect similar symptoms rather than showing that RLS and GP are the same condition.

However, a limited number of studies have speculated that they may represent a single condition. In particular, in 2022, Champion et al. (24) proposed that GPs are frequently misdiagnosed as painful RLS. They suggested refining the diagnostic criteria for GPs by adding an exclusion criterion related to the urge to move the legs—considered the hallmark symptom of RLS—to better define a more specific “GP-specific” phenotype. Their hypothesis encompassed two possibilities: (1) RLS and GPs are distinct conditions that may share common etiological mechanisms, or (2) GPs represent an early phenotypic expression of pediatric RLS, in which pain is predominant in younger children, while the urge to move and a decrease in pain become more prominent during adolescence. This would suggest that RLS and GPs may represent different developmental stages of the same condition. Despite the exclusion of the urge to move, GP- specific phenotype shares clinical similarities with RLS, particularly its painful variant, which is characterized by both an urge to move the legs and pain.

#### 3.3.1 Diagnostic overlap and proposed exclusion criteria

Several studies in the past decade have highlighted commonalities between RLS and GP, emphasizing overlapping diagnostic criteria and challenges in differential diagnosis. Evans et al. (26) were the first to propose distinct subgroups of leg pain, distinguishing between GP, RLS, and a mixed phenotype. Based on these observations, certain exclusion criteria have been suggested to improve diagnostic accuracy and to reduce misdiagnosis.

Although not all formally studied, in Tables 2, 3 we summarized and compared the main common and distinct features for RLS and GP. While the RLS criteria are structured and include both inclusion and exclusion elements, the diagnostic features for GP remain more variable and uncertain. Moreover, GP lacks standardized exclusion criteria. Key points of overlap between the two conditions include onset between ages 3 and 12, bilateral muscle pain or unpleasant sensations that worsen in the evening or at night, intermittent symptoms often relieved by gross movements, and the absence of functional limitations or objective findings. However, criteria such as the urge to move the legs, symptom relief through movement, possible unilateral symptoms, involvement of other body parts and presence of symptoms in the morning are more characteristic of RLS and help distinguish it from GP. These distinctions are critical for accurate diagnosis and management, yet they are not always clearly applied in clinical practice. A clearer definition of GP, including validated diagnostic and exclusion criteria, is needed to improve differentiation from RLS, especially in borderline or mixed presentations.

**Table 2:** Shared clinical manifestations of restless legs syndrome (RLS) and growing pains (GP).

Symptoms
Symptoms get worse during evening or night
Pain in both legs
Unpleasant sensations experienced like pain
The unpleasant sensations are relieved by gross movements such as stretching or walking around
Symptoms are felt in the muscles and not in the joints
No limitation of activity, no limping and no lack of well being
The pain/urge to move and the unpleasant sensations are intermittent with variable periods free from symptoms
Age of onset: 3–12 years

**Table 3:** Distinctive clinical features unique to restless legs syndrome (RLS) and growing pains (GP).

Symptoms	RLS	GP
Urge to move the legs (movement partially or totally relieves the unpleasant sensation) not necessarily accompanied by pain	x	
Symptoms can occur both at rest or during movement/physical activity		x
Possible involvement of arms or other body parts	x	
Symptoms can be unilateral	x	
The urge to move the legs and unpleasant sensations can be present in the morning	x	
No pain in the morning		x

#### 3.3.2 Association between RLS, GP, and headache

Beyond their potential relationship, RLS and GP frequently co-occur at rates higher than expected by chance alone. Donnelly et al. (25) found significant associations between persistent pain and multiple pain disorders, including GP, migraine, non-migraine headache, and recurrent abdominal pain.

The relationship between RLS, GP, and primary headaches has been extensively investigated. Several studies (22, 31, 32) have reported a higher prevalence of RLS among children with migraine and tension-type headache (TTH) compared to those without headaches. Associations with headache equivalents, such as recurrent abdominal pain, have also been noted (25, 28). Furthermore, Sevindik et al. (22) found that pediatric migraine patients with RLS experienced more frequent arousals than those without RLS, suggesting that this comorbidity may exacerbate sleep disturbances and contribute to increased migraine frequency.

While GP has also been proposed as part of the “migraine spectrum,” evidence specifically comparing the types and prevalence of headaches associated with GP or RLS remains limited (4, 8). Additional comparative research is needed to determine whether headache prevalence or type differs meaningfully between RLS and GP populations.

#### 3.3.3 Sleep disturbances in RLS and GP

The association between RLS, GP, and sleep disturbances has been a key focus of research. Further evidence supporting a connection between RLS and GP comes from polysomnographic (PSG) studies. Wong et al. (33) analyzed a cohort of children undergoing PSG and observed that children with GP were three times more likely to exhibit a Periodic Limb Movements of Sleep (PLMS) index ≥5/h than those without GP. PLMS is characterized by periodic, stereotyped lower limb movements, which may lead to arousals or awakenings, often without the individual’s awareness. While PLMS is a supportive diagnostic criterion for RLS, it can also occur in other sleep disorders, neurodegenerative diseases, and various medical conditions, including cardiovascular disease, chronic kidney disease, and depression. These findings suggest a potential link between GP and PLMS, reinforcing the hypothesis that GP might be part of the phenotypic spectrum of RLS.

Moraleda-Cibrián et al. (34) further explored this relationship in children with non-syndromic cleft palate, reporting that two-thirds of those with RLS symptoms screened positive for PLMS, whereas only 40% of children with PLMS reported RLS symptoms. Interestingly, 85.7% of children with GP symptoms also screened positive for PLMS, but only 14.3% of them reported RLS symptoms. These findings suggest that while PLMS is common in both conditions, its presence alone does not necessarily indicate RLS.

Although not the primary focus of this review, some polysomnographic studies comparing pediatric and adult RLS patients provide context for understanding the condition’s variability. Kim et al. (23) found that pediatric RLS presents with a more equal sex distribution, lower ferritin levels, less severe symptoms, and overall better sleep quality than adult RLS. Notably, PLMS was less frequently observed in pediatric RLS cases compared to adult RLS. Similarly, Yalcinkaya et al. (3) demonstrated that the prevalence of RLS is significantly higher (22%) among pediatric patients with PLMS compared to the estimated general pediatric prevalence of 2–4% suggesting a possible pathophysiological link between PLMS and pediatric RLS. However, the presence and diagnostic relevance of PLMS in GP remain unclear.

#### 3.3.4 Association with other medical comorbidities

Several studies have explored the association between RLS, GP, and various medical conditions. Angriman et al. (32) reported that RLS symptoms are linked to GP, diabetes, multiple sclerosis, chronic kidney disease, and liver transplantation. Similarly, Evans et al. (26) identified increased joint mobility, greater ankle dorsiflexion strength, and anemia as common predictors of both RLS and GP, with anemia present in 13% of all leg pain subgroups. Moreover, increased body weight, waist circumference, and BMI were all associated with leg pain.

Yalcinkaya et al. (3) highlighted a significantly higher prevalence of RLS in pediatric-onset multiple sclerosis (POMS) patients, whereas no correlation between GP and multiple sclerosis was identified in the literature.

The relationship between RLS, GP, and congenital conditions has also been investigated. Moraleda-Cibrián et al. (34) observed a high prevalence of RLS and GP symptoms in young children diagnosed with non-syndromic cleft palate. They hypothesized that these sleep-related disturbances could partially contribute to the cognitive and behavioral difficulties frequently reported in this population. However, no association was found between sleep-disordered breathing (SDB) and either RLS or GP symptoms.

A prospective cohort study started in 2021 by Wang et al. (35) aims to determine whether RLS is associated with an increased risk of cardiovascular and cerebrovascular diseases in a large adult population; however, results are not yet available because the follow-up period is scheduled to continue until December 2025.

#### 3.3.5 Neuropsychiatric comorbidities

The association between RLS, GP, and neuropsychiatric disorders has been widely explored. Angriman et al. (32) found that RLS symptoms were strongly associated with ADHD, with 42.9% of cases presenting RLS symptoms and 7.1% meeting the diagnostic criteria for RLS. Additional associations were observed with conduct disorders and Tourette syndrome/tics. Notably, a bidirectional relationship between RLS and depression was also reported. In contrast, GP was primarily associated with conduct disorders, without evidence of a direct link to ADHD or mood disorders.

Moraleda-Cibrián et al. (34) further examined the impact of RLS and GP on behavioral and psychological outcomes in children with non-syndromic cleft palate. Their findings suggested that children with RLS were more likely to experience daytime sleepiness, externalizing behaviors, psychiatric symptoms, and somatic complaints. Additionally, prolonged sleep latency in these children was associated with internalizing symptoms. Conversely, GP was linked to externalizing symptoms but did not appear to influence bedtime or daytime behavior.

### 3.4 Treatment options

The therapeutic approach to GP and RLS has been explored in several studies, with some treatments showing efficacy in both conditions.

In 2013, Walters et al. (11) suggested that dopamine agonists and alpha-2 ligand anticonvulsants, commonly used for RLS, could also be effective in GP. More recently, Angriman et al. (32) emphasized the importance of iron supplementation as a primary therapeutic strategy for childhood RLS, particularly in patients with ADHD and low serum ferritin levels. In fact, due to iron’s key role in dopamine synthesis, iron deficiency may contribute to the shared physiopathology between ADHD and RLS, with more severe deficiency linked to more significant symptoms. Studies show low ferritin levels in ADHD and RLS patients, but the precise relationship between ferritin levels and the presence of both conditions is still debated. The shared genetic factors, often indicated by a family history of RLS in children with ADHD, along with neuropathological features such as dopaminergic dysfunction and iron metabolism issues, suggest a connection between the two disorders (36, 37).

Although dopaminergic medications are the most commonly used first-line treatment for RLS, prolonged use may result in augmentation. As alternatives, other therapies such as opioids, glutamatergic agents, adenosine-based treatments, and sleep aids have also been explored (38).

Although there are no specific treatments for migraine equivalents such as GPs, the same medications used for the acute and preventive treatment of migraine, such as 5-HT precursors, can be applied to migraine equivalents, as they share the same underlying pathophysiological mechanisms (8). On the other hand, serotonin has a dual role in RLS. While increased serotonin transmission, as seen with the use of certain antidepressants, can worsen RLS symptoms, serotonin also plays a role in sleep regulation, potentially offering benefits when its function is balanced (39).

In a review from 2023, Cederberg et al. (39) examined the potential benefits of vitamin D supplementation in the management of RLS. While the findings were not entirely consistent, the authors highlighted that vitamin D deficiency is common among RLS patients and recommended that assessing and correcting vitamin D levels should be part of standard care (39). Similarly, a 2015 study (40) reported a high prevalence of vitamin D deficiency in children with GP. In this study, vitamin D supplementation significantly reduced pain intensity, suggesting that vitamin D therapy may be a beneficial intervention for alleviating pain in children with GP (40).

In addition to pharmacological treatments, novel non-invasive neuromodulation techniques have been investigated. Dirks et al. (41) reviewed the potential role of transcutaneous spinal direct current stimulation (tsDCS) as a promising alternative therapy for RLS, though further studies are needed to confirm its efficacy.

Evans et al. (26) highlighted the effectiveness of leg muscle stretching for growing pains (GP), as demonstrated in a randomized controlled trial from 1988. However, despite this evidence, stretching exercises are rarely recommended in clinical practice. The authors also stressed the need for further research on factors such as foot arches, foot strength, joint mobility, and deficiencies in vitamin D and iron before considering systemic interventions.

Similarly, for restless legs syndrome (RLS), non-pharmacological approaches - including massage, stretching, walking, cognitive distraction, and warm baths - can provide symptom relief, particularly in mild cases. However, their benefits are typically short-lived, and strong evidence supporting their efficacy remains limited. These strategies may still serve as useful adjuncts, potentially reducing reliance on higher doses of medication (42).

## 4 Discussion

RLS and GP are common conditions in the pediatric population, but their diagnosis remains challenging due to symptom overlap and the lack of universally accepted diagnostic criteria for GP. This overlap offer complicates the differential diagnosis. The question is still open: can GP and RLS be considered as two separate entities or not? Although RLS and GP are generally considered distinct entities, there are clear similarities between the two, suggesting the possibility of a connection. In particular, the nature of their symptoms, their timing and their episodic pattern highlight similarities that deserve further exploration. The relationship between RLS and GP may reflect different stages of the same condition, with growing pains representing a precursor to RLS, where pain predominates in younger children, and the urge to move the legs emerges during adolescence as the picture evolves.

There is growing evidence supporting the hypothesis of shared biological mechanisms between the two conditions. Dysfunctions in the serotoninergic system, alterations in iron metabolism and vitamin D deficiency have been implicated in both RLS and GP. These common factors suggest that similar neurochemical pathways may contribute to the development of symptoms in both disorders. Moreover, genetic studies suggest a familial predisposition to develop either RLS or GP. However, while RLS has been associated with specific gene variants involved in neural development and dopaminergic signaling, such associations have not been identified for GP. This suggests that, although both disorders may share a genetic predisposition, the underlying molecular mechanisms seem to differ.

Regarding diagnosis, it is important to consider the distinctive characteristics of each condition, in order to improve diagnostic accuracy and avoid misclassification. One of the key challenges in differentiating GP from RLS lies in the absence of standardized diagnostic criteria for GP.

According to our research, for RLS, pain without an associated urge to move the legs, lack of relief through movement (except in severe cases), and symptom onset exclusively during activity rather than at rest can help differentiate it from GP. Conversely, GP can be distinguished from RLS when pain is accompanied by a strong urge to move the legs or occurs in the morning. In addition, symptoms attributable to another medical or behavioral condition, the presence of abnormal physical examination or laboratory findings, or significant functional limitations (such as limping and associated reduced wellbeing) should prompt consideration of alternative diagnoses beyond RLS and GP.

As for treatment, both conditions benefit from similar therapeutic approaches. These include iron supplementation, serotonin, vitamin D and non-pharmacological interventions such as physical therapies like stretching or massage to alleviate symptoms.

The studies we chose to analyze in this review have shown that RLS and GP present many overlapping features, but at the same time, they are characterized by associations with various medical and psychiatric comorbidities, different genetic pathways, and distinct pathophysiology.

Despite the progress made in recent years, several important questions remain unanswered. According to our findings, further research should be performed in order to fully understand the nature of the relationship between the two conditions.

For example, no prospective longitudinal studies were found in literature, as well as studies regarding the circadian rhythm, as well as the role of vitamin D. Another critical gap in literature is the absence of validated diagnostic criteria for GP. This lack needs to be solved in order to improve diagnostic accuracy and prevent errors. Genetic and familial studies could be essential in determining whether there is a shared genetic predisposition between the two conditions, as well as the use of neurological imaging techniques, such as MRI, to identify any biological differences.

In conclusion, our review contributes to a clearer and more consistent characterization of the distinctive clinical features of GP, while underscoring the need for specific, evidence-based diagnostic and exclusion criteria for both GP and RLS. Although some overlapping features are observed, but the currently available evidence is insufficient to determine definitively whether GP and RLS are distinct disorders or manifestations of a shared underlying process. Therefore, based on the current literature, we conclude that no definitive classification can be established at this time.

Despite the important progress made over the past 10 years in understanding the differences and the overlapping characteristics of the two conditions, further studies are needed to address this unresolved issue. Advancing knowledge in this area will enhance diagnostic accuracy, improve patient outcomes, and support the development of more targeted therapeutic strategies for the pediatric population.

## References

[1] Walters AS. Toward a better definition of the restless legs syndrome. The international restless legs syndrome study group. Mov Disord. (1995) 10:634–42. doi: 10.1002/mds.870100517, PMID: 8552117

[2] Allen RP Picchietti DL Garcia-Borreguero D Ondo WG Walters AS Winkelman JW. International restless legs syndrome study group. Restless legs syndrome/Willis-Ekbom disease diagnostic criteria: updated international restless legs syndrome study group (IRLSSG) consensus criteria--history, rationale, description, and significance. Sleep Med. (2014) 15:860–73. doi: 10.1016/j.sleep.2014.03.025, PMID: 25023924

[3] Yalcinkaya BC Şahin S Gücüyener K Yüceyar N Yilmaz S. Restless legs syndrome in pediatric onset multiple sclerosis. Mult Scler Relat Disord. (2021) 56:103295. doi: 10.1016/j.msard.2021.103295, PMID: 34624645

[4] Tarantino S Capuano A Torriero R Citti M Vollono C Gentile S. Migraine equivalents as part of migraine syndrome in childhood. Pediatr Neurol. (2014) 51:645–9. doi: 10.1016/j.pediatrneurol.2014.07.018, PMID: 25155656

[5] Abu-Arafeh I Gelfand AA. The childhood migraine syndrome. Nat Rev Neurol. (2021) 17:449–58. doi: 10.1038/s41582-021-00497-6, PMID: 34040231

[6] Duchamp R-G. Maladies de la croissance. Paris: Fain (1823).

[7] Ekbom KA. Growing pains and restless legs. Acta Paediatr Scand. (1975) 64:264–6. 1130184 10.1111/j.1651-2227.1975.tb03832.x

[8] Frattale I Ruscitto C Papetti L Ursitti F Sforza G Moavero R. Migraine and its equivalents: what do they share? A narrative review on common pathophysiological patterns. Life (Basel). (2021) 11:1392. doi: 10.3390/life11121392, PMID: 34947923 PMC8705894

[9] Evans AM Scutter SD. Prevalence of “growing pains” in young children. J Pediatr. (2004) 145:255–8. doi: 10.1016/j.jpeds.2004.04.045, PMID: 15289780

[10] Champion D Pathirana S Flynn C Taylor A Hopper JL Berkovic SF. Growing pains: twin family study evidence for genetic susceptibility and a genetic relationship with restless legs syndrome. Eur J Pain. (2012) 16:1224–31. doi: 10.1002/j.1532-2149.2012.00130.x, PMID: 22416025

[11] Walters AS Gabelia D Frauscher B. Restless legs syndrome (Willis-Ekbom disease) and growing pains: are they the same thing? A side-by-side comparison of the diagnostic criteria for both and recommendations for future research. Sleep Med. (2013) 14:1247–52. doi: 10.1016/j.sleep.2013.07.013, PMID: 24157095

[12] Yoganathan S Chakrabarty B. Epidemiology of pediatric restless leg syndrome. Sleep Med Clin. (2025) 20:183–92. doi: 10.1016/j.jsmc.2025.02.001, PMID: 40348530

[13] Baethge C Goldbeck-Wood S Mertens S. SANRA—a scale for the quality assessment of narrative review articles. Research integrity and peer. Review. (2019) 4:64. doi: 10.1186/s41073-019-0064-8 PMC6434870 30962953

[14] Sandyk R. L-tryptophan in neuropsychiatric disorders: a review. Int J Neurosci. (1992) 67:127–44. doi: 10.3109/00207459208994781 1305630

[15] Koo BB Bagai K Walters AS. Restless legs syndrome: current concepts about disease pathophysiology. Tremor Other Hyperkinet Mov. (2016) 6:401. doi: 10.7916/D83J3D2G, PMID: 27536462 PMC4961894

[16] Jhoo JH Yoon IY Kim YK Chung S Kim JM Lee SB. Availability of brain serotonin transporters in patients with restless legs syndrome. Neurology. (2010) 74:513–8. doi: 10.1212/WNL.0b013e3181cef824, PMID: 20142619

[17] Winlow W. Pramipexole in restless legs syndrome: an evidence-based review of its effectiveness on clinical outcomes. Core Evid. (2005) 1:35–42. doi: 10.2147/CE.S6405, PMID: 22496675 PMC3321653

[18] Guo S Huang J Jiang H Han C Li J Xu X. Restless legs syndrome: from pathophysiology to clinical diagnosis and management. Front Aging Neurosci. (2017) 9:171. doi: 10.3389/fnagi.2017.00171, PMID: 28626420 PMC5454050

[19] Silva-Néto RP Soares AA Souza WPO Krymchantowski AG Jevoux C Krymchantowski A. "Growing pains" in children and adolescents as an early symptom of migraine: a prospective study. Headache. (2023) 63:1070–5. doi: 10.1111/head.14608, PMID: 37671464

[20] Panconesi A. Serotonin and migraine: a reconsideration of the central theory. J Headache Pain. (2008) 9:267–76. doi: 10.1007/s10194-008-0058-2, PMID: 18668197 PMC3452194

[21] Hashkes PJ Friedland O Jaber L Cohen HA Wolach B Uziel Y. Decreased pain threshold in children with growing pains. J Rheumatol. (2004) 31:610–3. PMID: 14994414

[22] Sevindik MS Demirci S Göksan B Özge A Savrun FK Onur H. Accompanying migrainous features in pediatric migraine patients with restless legs syndrome. Neurol Sci. (2017) 38:1677–81. doi: 10.1007/s10072-017-3045-z 28669082

[23] Kim S Kim KT Motamedi GK Cho YW. Clinical characteristics of Korean pediatric patients with restless legs syndrome. Sleep Med. (2020) 69:14–8. doi: 10.1016/j.sleep.2020.01.002 32045850

[24] Champion GD Bui M Sarraf S Donnelly TJ Bott AN Goh S. Improved definition of growing pains: a common familial primary pain disorder of early childhood. Paediatr Neonatal Pain. (2022) 4:78–86. doi: 10.1002/pne2.12067 35719219 PMC9189907

[25] Donnelly TJ Bott A Bui M Goh S Jaaniste T Chapman C. Common pediatric pain disorders and their clinical associations. Clin J Pain. (2017) 33:1131–40. doi: 10.1097/AJP.0000000000000490 28272118

[26] Evans AM Berde T Karimi L Ranade P Shah N Khubchandani R. Correlates and predictors of paediatric leg pain: a case–control study. Rheumatol Int. (2018) 38:835–43. doi: 10.1007/s00296-018-3983-x 29797060

[27] Türkdoğan D Mahmudov R. Overlapping features of restless legs syndrome and growing pains in Turkish children and adolescents. Brain Dev. (2022) 44:372–9. doi: 10.1016/j.braindev.2022.02.005 35221168

[28] Champion D Pathirana S Flynn C Taylor A Hopper JL Berkovic SF. Contrasting painless and painful phenotypes of pediatric restless legs syndrome: a twin family study. Sleep Med. (2020) 75:361–7. doi: 10.1016/j.sleep.2020.08.024 32950881

[29] Catoire H Sarayloo F Mourabit Amari K Apuzzo S Grant A Rochefort D. A direct interaction between two restless legs syndrome predisposing genes: MEIS1 and SKOR1. Sci Rep. (2018) 8:12173. doi: 10.1038/s41598-018-30665-6, PMID: 30111810 PMC6093889

[30] Sehgal A Mignot E. Genetics of sleep and sleep disorders. Cell. (2011) 146:194–207. doi: 10.1016/j.cell.2011.07.004, PMID: 21784243 PMC3153991

[31] Seidel S Böck A Schlegel W Kilic A Wagner G Gelbmann G. Increased RLS prevalence in children and adolescents with migraine: a case-control study. J Headache Pain. (2012) 32:693–9. doi: 10.1177/0333102412446207, PMID: 22659118

[32] Angriman M Cortese S Bruni O. Somatic and neuropsychiatric comorbidities in pediatric restless legs syndrome: a systematic review of the literature. Sleep Med Rev. (2017) 34:34–45. doi: 10.1016/j.smrv.2016.06.006 27519964

[33] Wong MW Williamson BD Qiu W Champion D Teng AJ. Growing pains and periodic limb movements of sleep in children. Paediatr Child Health. (2014) 50:455–60. doi: 10.1111/jpc.12493, PMID: 24547979

[34] Moraleda-Cibrián M Edwards S Kasten S Warschausky S Buchman S O'Brien L. Sleep-related movement disorders and growing pains: differences in daytime and bedtime behavior in 2-6 year old children with cleft palate. Sleep Med. (2021) 85:303–8. doi: 10.1016/j.sleep.2021.06.027 34391005

[35] Wang SH Chen XY Wang XP. Jidong restless legs syndrome cohort study: objectives, design, and baseline screening. Front Neurol. (2021) 12:682448. doi: 10.3389/fneur.2021.682448, PMID: 34721252 PMC8548385

[36] Montoya AC Mesa SC Cuartas JM Ochoa WC. Prevalence and clinical characteristics of the restless legs syndrome (RLS) in patients diagnosed with attention-deficit hyperactivity disorder (ADHD) in Antioquia. Int J Psychol Res. (2018) 11:58–69. doi: 10.21500/20112084.3381, PMID: 32612771 PMC7110177

[37] Konofal E Cortese S Marchand M Mouren MC Arnulf I Lecendreux M. Impact of restless legs syndrome and iron deficiency on attention-deficit/hyperactivity disorder in children. Sleep Med. (2007) 8:711–5. doi: 10.1016/j.sleep.2007.04.022 17644481

[38] Lv Q Wang X Asakawa T Wang XP. Pharmacologic treatment of restless legs syndrome. Curr Neuropharmacol. (2021) 19:372–82. doi: 10.2174/1570159X19666201230150127, PMID: 33380302 PMC8033969

[39] Cederberg KLJ Silvestri R Walters AS. Vitamin D and restless legs syndrome: a review of current literature. Tremor Other Hyperkinet Mov. (2023) 13:741. doi: 10.5334/tohm.741, PMID: 37034443 PMC10077981

[40] Vehapoglu A Turel O Turkmen S Inal BB Aksoy T Ozgurhan G. Are growing pains related to vitamin D deficiency? Efficacy of vitamin D therapy for resolution of symptoms. Med Princ Pract. (2015) 24:332–8. doi: 10.1159/000431035, PMID: 26022378 PMC5588252

[41] Dirks CAH Bachmann CG. From brain to spinal cord: neuromodulation by direct current stimulation and its promising effects as a treatment option for restless legs syndrome. Front Neurol. (2024) 15:1278200. doi: 10.3389/fneur.2024.1278200, PMID: 38333606 PMC10850250

[42] Gossard TR Trotti LM Videnovic A St Louis EK. Restless legs syndrome: contemporary diagnosis and treatment. Neurotherapeutics. (2021) 18:140–55. doi: 10.1007/s13311-021-01019-4, PMID: 33880737 PMC8116476

